# Ketogenic Diet and Epilepsy

**DOI:** 10.3390/nu11102510

**Published:** 2019-10-18

**Authors:** Marzena Ułamek-Kozioł, Stanisław J. Czuczwar, Sławomir Januszewski, Ryszard Pluta

**Affiliations:** 1 Laboratory of Ischemic and Neurodegenerative Brain Research, Mossakowski Medical Research Centre, Polish Academy of Sciences, 02-106 Warsaw, Poland; mulamek@imdik.pan.pl (M.U.-K.); sjanuszewski@imdik.pan.pl (S.J.); 2 First Department of Neurology, Institute of Psychiatry and Neurology, 02-957 Warsaw, Poland; 3 Department of Pathophysiology, Medical University of Lublin, 20-090 Lublin, Poland; czuczwarsj@yahoo.com

**Keywords:** epilepsy, drug-resistant epilepsy, ketogenic diet, therapy, ketones, gut microbiota, side effects

## Abstract

Currently available pharmacological treatment of epilepsy has limited effectiveness. In epileptic patients, pharmacological treatment with available anticonvulsants leads to seizure control in <70% of cases. Surgical intervention can lead to control in a selected subset of patients, but still leaves a significant number of patients with uncontrolled seizures. Therefore, in drug-resistant epilepsy, the ketogenic diet proves to be useful. The purpose of this review was to provide a comprehensive overview of what was published about the benefits of ketogenic diet treatment in patients with epilepsy. Clinical data on the benefits of ketogenic diet treatment in terms of clinical symptoms and adverse reactions in patients with epilepsy have been reviewed. Variables that could have influenced the interpretation of the data were also discussed (e.g., gut microbiota). The data in this review contributes to a better understanding of the potential benefits of a ketogenic diet in the treatment of epilepsy and informs scientists, clinicians, and patients—as well as their families and caregivers—about the possibilities of such treatment. Since 1990, the number of publications on attempts to treat drug-resistant epilepsy with a ketogenic diet has grown so rapidly that it has become a challenge to see the overall trajectory and major milestones achieved in this field. In this review, we hope to provide the latest data from randomized clinical trials, practice guidelines, and new research areas over the past 2 years.

## 1. Introduction

Epilepsy is a chronic brain disorder that is characterized by recurrent seizures, which are short episodes of involuntary movement that can affect part or all of the body, sometimes accompanied by loss of consciousness and control of bladder or bowel function. Epilepsy is defined as the occurrence of 2 or more unprovoked seizures. A common type of epilepsy affecting 6 in 10 people is idiopathic epilepsy, which means that in over 50% of global cases, the cause of the disease is not identified [1]. Epilepsy of known cause is called secondary or symptomatic epilepsy. Causes of secondary or symptomatic epilepsy are: brain tumors, stroke, brain infection and severe head injury, congenital abnormalities associated with brain defects, brain damage as a result of prenatal or perinatal injuries, and certain genetic syndromes [2]. About 50–70 million people worldwide suffer from epilepsy [2,3]. It is estimated that 2.4–4.6 million people worldwide are diagnosed with epilepsy each year [3]. These global load estimates are falling more on the populations of low- and middle-income countries, where the cumulative estimate of annual incidence of epilepsy is much higher (139 per 100,000 people) than in high-income countries (49 per 100,000 people) [3]. Regardless of the country’s income, the public health burden of epilepsy carries a high risk of disability, economic loss, social isolation, and premature death [4]. Epilepsy is a serious and costly health problem worldwide and includes estimated indirect and direct costs annually of around EUR 15.5 billion in Europe [4] and USD 15.5 billion in the United States [5]. In this regard, the World Health Organization has made this a priority, calling for the development of national healthcare plans for the treatment of epilepsy, not only to ensure the availability of effective care, but also to prevent its causes. Almost 80–90% of people diagnosed with epilepsy live in low- and middle-income countries [2,6]. Recent studies in low-, middle-, and high-income countries have shown that up to 70% of adults and children with epilepsy can be successfully treated with antiepileptic drugs. After 2 to 5 years of successful therapy and no seizures, medications can be withdrawn in approximately 70% of children and 60% of adults without recurrence. As the above information shows, available pharmacological treatment for epilepsy has limited effectiveness. Surgical intervention can lead to seizure control in a selected subset of patients, but still leaves a significant number of patients with uncontrolled seizures. The ketogenic diet has proven useful in cases of epilepsy in which pharmacological and/or surgical treatment is not effective as shown below. The purpose of this review was to provide a comprehensive overview of what was published about the benefits of ketogenic diet treatment in patients with epilepsy. Clinical data on the benefits of ketogenic diet treatment in terms of clinical symptoms and adverse reactions in patients with epilepsy have been reviewed. Variables that could have influenced the interpretation of the data were also discussed. The data in this review contributes to a better understanding of the potential benefits of a ketogenic diet in the treatment of epilepsy and informs scientists, clinicians, and patients, as well as their families and caregivers, about the possibilities of such treatment. Since 1990, the number of publications on attempts to treat drug-resistant epilepsy with a ketogenic diet has grown so rapidly that it has become a challenge to see the overall trajectory and major milestones achieved in this field. In this review, we hope to provide the latest data from randomized clinical trials, practice guidelines, and new research areas over the past 2 years.

## 2. Classic Epilepsy Therapy

Classic epilepsy treatment includes pharmacological and surgical therapy or vagus nerve stimulation. Despite these therapies, approximately 30% of patients with epilepsy do not have sufficiently controlled seizures and become resistant to drugs [7]. This is defined as insufficient seizure control, despite optimal therapy using a combination of two or more appropriately selected antiepileptic drugs. Under these circumstances, adding next antiepileptic drug often does not significantly reduce seizures. Although epilepsy research is ongoing, the mechanisms of this disease have not been completely elucidated and fully effective therapy for all epilepsy patients has not yet been developed. Epilepsy is the highest research priority for many pharmaceutical companies, which makes epilepsy one of the most studied brain disease in the pharmaceutical industry, but despite such tremendous commitment, we are not seeing significant progress in developing new effective drugs. Patients with drug-resistant epilepsy are addicted to informal care of family and friends as well as healthcare professionals such as social workers, neurologists, and psychologists. Problems associated with drug-resistant epilepsy in children, adolescents, and adults cause repeated hospitalizations of numerous patients. Living with uncontrolled epilepsy has a negative impact on the quality of life of patients with epilepsy and their caregivers.

## 3. Ketogenic Diet

A ketogenic diet should be considered for patients who have not responded adequately to therapy with two well-selected and well-dosed antiepileptic drugs. Therefore, neurologists often recommend other therapies, such as diet, including ketogenic diet, to provide patients with better antiepileptic control. The ketogenic diet is a last resort treatment for many children, adolescents, and adults with epilepsy resistant to routine medications. It should be recognized that, despite the development of new antiepileptic drugs every year, the treatment, as already mentioned, in about one-third of patients with epilepsy is not fully effective. Ketogenic diet treatment is a non-pharmacological therapy used worldwide, especially for children with epilepsy that is difficult to control. Ketogenic diet has been used in patients with difficult-to-treat epilepsy since 1921, with minor changes in recent years [7]. The ketogenic diet assumes a very high-fat and low-carbohydrate diet, reducing carbohydrate to less as 10% of used energy [8]. This restriction triggers a systemic shift from glucose metabolism toward the metabolism of fatty acids yielding ketone bodies, such as acetoacetate and β-hydroxybutyrate as substrates for energy. The ketogenic diet provides sufficient protein for growth and development. Energy is mostly derived from fat delivered in the diet and by the utilization of body fat. The ketogenic diet is a biochemical model of fasting, which shifts organs to utilize ketone bodies as the source to replace glucose for the brain. The ketogenic diet allows about 90% of the total caloric income from fat and 6% from protein and 4% from carbohydrates. For many refractory epileptic patients, dietary treatment promises to improve the quality of life with a significant decrease in seizure frequency. For this reason, an increase in the global use of the ketogenic diet is currently observed. Successful implementation of this diet depends on the active support of the health care team, the social and educational system, and finally the family. The ketogenic diet requires strict dietary and medical control due to its restrictiveness and side effects [6,7].

## 4. A Brief History of Ketogenic Diet Treatment and Its Side Effects 

In 1998, a multicenter study was conducted in 51 children with drug-resistant epilepsy [9]. Forty-seven % of children remained on a diet for a year. Forty-three % of them were seizure-free, 39% controlled 50–90% of seizures, and 17% did not respond. Adverse reactions associated with the administration of a ketogenic diet were severe dehydration or acidosis, lethargy, somnolence, severe infections, mood swings, vomiting, and constipation. The reasons for discontinuing treatment were intolerance, difficulties in maintaining a restrictive diet and inadequate seizure control. The authors thought that the decrease in seizures was unlikely to be a placebo effect [9].

In the same year, a group from Johns Hopkins published a study conducted on 150 children aged 1–16 years [10]. One year after starting the ketogenic diet, 7% were without seizures, 27% of children had a decrease in seizure frequency >90% and 50% a seizure frequency reduction >50%. The authors noted that children which after a ketogenic diet had reduction in seizure frequency >50% during the first three months of treatment resulted in the gradual improvement during prolong therapy, but if no 50% seizure reduction was observed at that time, it was unlikely that improvement would occur in the following months [10,11].

Seventy children with drug resistant epilepsy were qualified for a retrospective long-term study at the University of São Paulo to assess the effectiveness and tolerability of the ketogenic diet [12]. Within one year, in 55% of those who remained on the ketogenic diet, 70% had seizure control >75%, 25% had seizure control in the range of 50–75%, and 2.5% had seizure control <50%. The effectiveness of the ketogenic diet was significantly higher in cases of generalized epilepsy than partial epilepsy. About 10% of children discontinued their diet due to distaste, and 3.7% experienced vomiting and nausea [12].

The Neal’s group in 2008 [13] conducted the first randomized clinical controlled trial to assess the effectiveness of a ketogenic diet in drug-resistant epilepsy. They observed 145 children with epilepsy who did not respond to two antiepileptic drugs. The children were randomly divided into two groups: one received a ketogenic diet immediately, and the other after three months with a combination of two antiepileptic drugs. After three months, the ketogenic dietary group had a 75% reduction in seizure frequency compared to the control group. In addition, 38% of children in the ketogenic diet group had a >50% reduction in seizures, and 7% had a >90% decrease in seizure frequency. The data showed that the ketogenic diet has advantages over no change in treatment. Almost 25% of children treated with the ketogenic diet reported side effects such as lack of energy, vomiting, hunger, abdominal pain, diarrhea, and taste problems. However, the most commonly reported side effect of ketogenic diet treatment was constipation [13].

A ketogenic diet can also be a reasonable alternative for adults with difficult-to-treat epilepsy, providing a fast, reversible option to the vagus nerve or deep brain stimulation. The published meta-analysis evaluated 270 patients, of whom 168 were given a ketogenic diet [14]. The rates of effectiveness of a ketogenic diet in difficult-to-treat epilepsy for adults ranged from 13 to 70%. The meta-analysis showed a combined ketogenic diet efficiency rate of 42%, with significant heterogeneity in all studies. In addition, the data indicated that the ketogenic diet can be a promising complement to incurable epilepsy therapy in adults and is best started with a modified Atkins diet and then consider switching to the classic ketogenic diet if greater seizure control is required [14]. To support the above suggestions, well-organized and controlled clinical trials in adults are necessary.

In addition, the ketogenic diet is the therapy of choice for GLUT1 deficiency syndrome and pyruvate dehydrogenase deficiency [15,16]. In both diseases, the ketogenic diet provides ketones that bypass metabolic defects and serve as an alternative fuel for the brain [17]. In myoclonic epilepsy, including infancy myoclonic epilepsy (Dravet’s syndrome), and especially myoclonic-atonic epilepsy (Doose’s syndrome), ketogenic diet appears to be effective in leading to seizure freedom and may need to be considered at an earlier stage of treatment [15,16].

The Pires’ group [18] demonstrated the effectiveness of a ketogenic diet in the treatment of infantile spasms as a third-line therapy after vigabatrin and steroids. After 1 month, 35% of patients had no seizures and as many as 65% had no seizures after the third month of treatment. It should be emphasized, however, that after a month patients without seizures received additional new anti-epileptic drugs. The effectiveness of the ketogenic diet remained stable for 3 to 6 months, and the diet was not interrupted due to tolerance [18].

Recently several randomized clinical trials have been published that support the use of a ketogenic diet in the treatment of drug-resistant epilepsy, but these studies have a weak point associated with small sample sizes. For example, a randomized controlled study of a ketogenic diet in refractory pediatric epilepsy was studied for 4 months in 48 children (26 ketogenic diet, 22 care as usual) aged 1–18 years [19]. The average seizure frequency after 4 months compared to baseline was significantly lower in the ketogenic diet group (56%) than in the care as usual group (99%). Twice as many patients in the dietary ketogenic group had a significant decrease in seizure frequency. Patients treated with a ketogenic diet, however, had a significantly higher result in gastrointestinal symptoms [19]. 

Martin-McGill et al. [20] published an updated Cochrane review on the evidence for ketogenic diet anti-seizure activity from randomized clinical trials. In randomized clinical trials, 778 patients participated in 11 trials; 712 children and adolescents and 66 adults. Reported seizure freedom rates ranged from 0 to 55% after three months, and reported seizure reduction rates reached up to 85%. One trial found no significant difference between the fasting-onset and gradual onset ketogenic diet of seizure freedom, and a greater seizure reduction rate was found in the gradual-onset ketogenic diet group. All studies had adverse effects of dietary interventions. The most commonly reported adverse reactions were gastrointestinal syndromes. Side effects were the most common reason for participants dropping out of research. Other reasons for giving up were lack of effectiveness and lack of diet acceptance. One study assessed the impact of dietary interventions on quality of life, cognition and behavioral functioning, participants in the ketogenic diet study being more active, productive, and less anxious after four months compared with the control group. However, there was no significant difference in quality-adjusted years of life between the ketogenic dietary group and the control group after four or 16 months [20]. It should be borne in mind that these positive findings from studies spanning a decade, were obtained by different clinical groups in children with highly refractory epilepsy.

Other studies on the ketogenic diet in adults with drug-resistant epilepsy have provided good evidence of dietary efficacy [21]. This study reviewed 16 prospective studies involving 338 patients and found that the overall efficacy index for seizure freedom was 13%, with seizures reduced by more than 50%, exceeding 53% [21]. The results of the meta-analysis showed that the combined efficacy rates for all symptoms of freedom of seizure, reduction of seizures by 50% or more, and reduction of seizures below 50% in adults with difficult-to-treat epilepsy were 13%, 53%, and 27%, respectively [21]. The limitation was that the performance indicators were based only on analysis by protocol (i.e., those who completed the study) and not based on treatment intent. This introduces an obvious bias in these superiority studies, but it can also be concluded that the ketogenic diet is effective in adults with refractory epilepsy and is fairly well tolerated.

In the latest meta-analysis study in children and adolescents with refractory epilepsy after a classic ketogenic diet, the percentage of patients whose seizure reduction ≥50% was 62, 60, 52, 42, and 46% in 1, 3, 6, 12, and 24 months of diet, respectively [22].

## 5. Possible Anti-Seizure Mechanisms of the Ketogenic Diet

**Figure 1:**
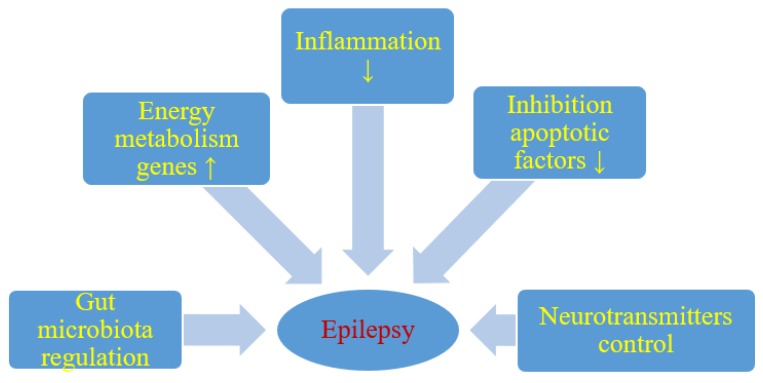
Likely effect of a ketogenic diet on seizure activity. ↑- increase, ↓- decrease.

Although the anticonvulsant mechanisms of ketogenic diet are not still completely understood, it is believed that ketone bodies and polyunsaturated fatty acids presumably play a major role in the anticonvulsant effect of ketogenic diet. During ketogenic diet treatment, body energy is generally generated by the oxidation of fatty acids in mitochondria, resulting in the production of large amounts of acetyl-CoA. Accumulation of acetyl-CoA leads to the synthesis of two ketone bodies mainly in the liver, acetoacetate, and β-hydroxybutyrate, which then enter the blood circulation. Ketone bodies are then used as an alternative source of energy in the brain instead of glucose. After entering the brain, the ketone bodies are transformed into acetyl-CoA and then enter the tricarboxylic acid cycle in the mitochondria of the brain, which ultimately leads to the production of adenosine triphosphate (ATP). Several hypotheses regarding ketone bodies are considered as key mediators involved in the anticonvulsant effect of the ketogenic diet. Based on several studies, potential mechanisms focus essentially on the role of neurotransmitters, brain energy metabolism, oxidative stress, and ion channels, which are briefly discussed below [23,24] (Figure 1).

It has been shown that energy production in the brain is significantly increased by ketogenic diet. Long-term ketogenic diet therapy increases the expression of energy metabolism genes, improves mitochondrial biogenesis and density, and increases energy reserves in the form of phosphocreatine [24] (Figure 1). This improves the function of neurons and increases their chances of surviving in stressful conditions. It is believed that brain tissue under the influence of a ketogenic diet becomes more resistant to metabolic stress, and this increases the seizure threshold. Under the conditions of a ketogenic diet, a decrease in brain glucose consumption and the production of glycolytic ATP may induce potassium channels sensitive to ATP opening, which leads to hyperpolarization of the neuronal membrane. This reduces electrical excitability of the brain and increases the seizure threshold. Additionally this prevents excessive firing of neurons and regulates the seizure threshold in the brain. It is also suggested that two-pore domain potassium channels may also be activated by ketone bodies and some fatty acids. Thus, a ketogenic diet triggered increase in the blood ketone bodies and fatty acids may also regulate neuronal membrane excitability by activating two-pore domain potassium channels, and this can be taken as another likely anticonvulsant mechanism of the ketogenic diet.

Reduction of neuronal excitability is the most important role of GABA in the brain and therefore GABA plays a key role in the initiation and spread of seizure activity in the brain. It has been observed that ketogenic diet can lead to glutamic acid decarboxylase activation, which induces GABA synthesis [23]. It has also been shown that this diet can alter GABA transaminase activity that inhibits GABA degradation. Increasing energy metabolism through a ketogenic diet can compensate for the metabolic and transient failure of GABAergic inhibition, the lack of which will not prevent the occurrence and spread of seizures. Therefore, another important mechanism induced by the ketogenic diet in anticonvulsant activity is probably mediated by the GABAergic system [23] (Figure 1).

High levels of glutamate in the brain can make the brain more susceptible to seizures and therefore glutamate is associated with the development of epilepsy. The results of studies on the effect of the ketogenic diet on glutamate level are inconclusive [24], it has been shown that this diet can increase the level of glutamate in the brain synaptosomes, while other studies showed no effect [23,25].

Agmatine has been found in synapses and can be considered as an inhibitory neurotransmitter. It may exert an anti-seizure effect, probably by inhibiting various brain stimulating receptors, including N-methyl-D-aspartate, histamine, and adrenaline receptors. It has been shown in rat studies that ketogenic diet can increase the level of agmatine in the hippocampus [23] (Figure 1). The above observations support the view that the ketogenic diet increases the level of agmatine in the brain, which has neuroprotective properties, therefore these properties can be considered as another anticonvulsant mechanism of the ketogenic diet [23]. Additionally, agmatine may potentiate the anticonvulsant action of valproate and phenobarbital against maximal electroconvulsions in mice with no pharmacokinetic interaction involved [26]. In another model of seizures induced by pentylenetetrazol in mice, agmatine was documented to attenuate the protective activity of vigabatrin, other numerous antiepileptic drugs being not affected [27]. If agmatine is involved in the mechanism of the ketogenic diet in clinical conditions, then some positive or negative outcomes may be observed when combined with particular antiepileptic drugs.

It has been noted that monoamine neurotransmitters—including noradrenaline, dopamine, and serotonin—play an important role in controlling the excitability of neurons and seizures [28,29]. Many neural networks have been shown to have serotonin and dopamine receptors that are involved in seizures. It has been shown that in animals without a functional noradrenergic system, the ketogenic diet did not show anticonvulsant capacity [24]. In addition, it has also been shown that the levels of serotonin and dopamine in the cerebrospinal fluid may be affected by ketogenic diet in children with drug-resistant epilepsy [29].

In addition, it appears that particularly polyunsaturated fatty acids provided by the ketogenic diet may activate peroxisome proliferator-activated receptors that regulate anti-inflammatory, antioxidant, and mitochondrial genes leading to increased energy reserves, stabilization of synaptic functions and restriction of hyperexcitability [30] (Figure 1).

It has been revealed that single small convulsions are not able to kill neurons, while severe long-term seizures can not only cause neuronal damage but also their death [24,31]. Cognitive impairment and severity of seizures in patients with drug-resistant epilepsy may depend on the degree of neuronal damage and death caused by seizures [32]. Everything indicates that excitotoxicity and apoptosis are the main mechanisms involved in seizure-related neuronal damage and death. It has been suggested that the negative consequences of these neuropathological processes can be improved by means of a ketogenic diet [33] (Figure 1). It has been observed that ketogenic diet can upregulate calbindin which has neuroprotective potential through its ability to buffer intracellular calcium [34]. Other neuroprotective properties of the ketogenic diet may mediate the inhibition of apoptotic factors such as caspase 3 [34,35] (Figure 1). Opening transient pores in mitochondria can also be inhibited by a ketogenic diet [36].

## 6. Ketogenic Diet and Gut Microbiota: Friends or Foes?

Dysbiosis may be involved in the drug-resistant epilepsy mechanism, and restoration of intestinal microbes may be a new therapeutic method in drug-resistant epilepsy [37,38,39] (Figure 1). People with drug-resistant epilepsy show altered intestinal microflora [37,38]. Numerous rare flora increases in patients with drug-resistant epilepsy [38]. Then Hampton [37] suggested that the antiepileptic effect of the ketogenic diet could be attributed to intestinal microbes (Figure 1).

The mechanisms by which the ketogenic diet exerts an anticonvulsant effect are likely to be numerous and may vary in different types of epilepsy. Recent articles describe a new mechanism for ketogenic diet to prevent seizures by changing gut microbiota in animals and humans [40,41,42]. To date, very few studies have focused on the role of gut microbiota in the treatment of epilepsy using a ketogenic diet [40,41,42,43]. Olson et al. [40], presented very interesting research on gut microbiota-dependent anticonvulsant properties of the ketogenic diet in which two mouse models of refractory epilepsy were used, demonstrating the relationship between the ketogenic diet and gut microbiota to obtain a therapeutic effect. Diet significantly increases the relative abundance of *Akkermansia muciniphila*, from 2.8% to 36.3% during 4 and 14 days of dietary treatment. *Parabacteroides merdae, Sutterella*, and *Erysipelotrichaceae* also increased significantly, while *Allobaculum, Bifidobacterium*, and *Desulfovibrio* were lower in mice fed the ketogenic diet compared to mice fed the control diet. *Akkermansia muciniphila* and *Parabacteroides merdae* have been shown both to be necessary to achieve the anti-seizure effect of a ketogenic diet. The combination of these two bacterial taxa restored protection against seizures in antibiotic-treated mice after administration of the ketogenic diet. On the contrary, colonization with only *Akkermansia muciniphila* or *Parabacteroides distasonis* did not protect against seizures and there was no significant increase in seizure threshold. Similarly, colonization of *Akkermansia muciniphila* and *Parabacteroides* together, but not separately, protected against seizures in germ-free mice fed a ketogenic diet [40].

Comparing mice protected against seizures and prone to attacks (diet, bacterial colonization, or antibiotic treatment), most metabolites that differentiate these groups were associated with amino acid metabolism, including lysine, tyrosine, and threonine derivatives [40]. In addition, there has been a common decline in ketogenic subgroups of γ-glutamylated amino acids in both the intestines and blood in mice protected from seizures compared to seizure-prone mice. Importantly, peripheral amino acids are used as substrates for the synthesis of γ-aminobutyric acid (GABA), an inhibitory neurotransmitter. The level of GABA in the hippocampus was higher compared to glutamate, a stimulating neurotransmitter, in mice fed the ketogenic diet compared to mice fed the control diet [40]. These increases were abolished in antibiotic-treated mice fed a ketogenic diet and restored after colonizing these mice with *Akkermansia muciniphila* and *Parabacteroides distasonis* [40].

Studies in humans have also shown changes in the intestinal microflora after a ketogenic diet, but bacterial changes varied depending on the report [41,42]. The study of the effect of the ketogenic diet on children with drug-resistant epilepsy showed a decrease in the diversity of the intestinal microflora after 1 week, along with a decrease in *Proteobacteria* and an increase in *Bacteroidetes* [44]. At the genus level, a decrease in *Cronobacter* was observed alongside increases in *Bifidobacterium*, *Bacteroides*, and *Prevotella*. The microflora in infants with epilepsy has been found to differ from healthy control and has been shown to change significantly, along with an increase in beneficial bacteria and a decrease in the number of pathogenic bacteria, in response to a ketogenic diet [44]. The study suggests that the ketogenic diet can quickly modify the intestinal microflora, which reduces the frequency of seizures in drug-resistant infants. In a separate study, the fecal microflora profiles of children with refractory epilepsy showed reduced diversity, with reduced *Firmicutes* and *Actinobacteria* levels and elevated levels of *Bacteroidetes* following 6 months of ketogenic diet treatment [41]. Participants in this study showed different responses to seizure reduction, and those who did not respond to treatment had elevated levels of *Alistipes*, *Clostridiales*, *Lachnospiraceae*, *Ruminococcaceae*, and *Rikenellaceae* in relation to those who responded positively to treatment [41]. This suggests that the ketogenic diet may have varying efficacy in changing the composition of the intestinal microflora, and that the specific microflora can provide both possible treatment targets and biomarkers for therapy efficacy in people with refractory epilepsy [41]. The study presented above was criticized by Spinelli and Blackford [45], in their opinion, the study raises some interesting questions: “1) How does gut bacteria affect epileptogenesis?, 2) Can monitoring gut bacterial composition be used as a marker for treatment efficacy and, as is seen in mouse models?, 3) Can altering bacterial composition be used as a therapeutic strategy?”. These opponents also point to the need to conduct a comprehensive, multicenter study based on the above observations and to better document whether possible microbiological therapy is a rational choice for refractory epilepsy in children [45].

Another recent study asked six people with GLUT1 deficiency syndrome to take stool samples before and following 3 months of ketogenic diet intake to compare the composition of the microflora [46]. *Bacteroidetes*, *Clostridium cluster XIV*, *Bifidobacterium* spp., *Clostridium perfringens*, *Enterobacteriaceae*, *Desulfovibrio* spp., *Faecalibacterium prausnitzii*, *Lactobacillus* spp., and *Firmicutes* were quantified by reverse transcription polymerase chain reaction. Fecal microbiological profiles showed a statistically significant increase in the level of *Desulfovibrio* spp., a group of bacteria that is likely to exacerbate inflammation intestinal mucosa as a result of animal consumption fat [46].

An interesting study on 12 children with drug-resistant epilepsy have recently been published [42]. Stool samples were taken before and following 3 months of taking the ketogenic diet, and the control group was parents. Changes in both taxonomic and functional profiles were detected by shotgun metagenomics DNA sequencing. Treatment resulted in a noticeable decrease in the relative abundance of *Dialister*, *Actinobacteria*, *Bifidobacteria*, and *Eubacterium rectale* together with a parallel increase in the relative abundance of *Escherichia coli* [42]. The study expressed concerns about the effects of ketogenic diet on the gut microflora and the general health of patients, as the relative numbers of beneficial fiber-consuming bacteria decreased in response to ketogenic diet treatment [42]. In the research of Tagliabue et al. [43], no changes in *Bacteroides* and *Firmicutes* was noted but significant increase in *Desulfovibrio* spp., were found following the ketogenic diet in children.

## 7. Conclusions

The randomized clinical trials presented in this review show promising results in the use of ketogenic diet in drug-resistant epilepsy. However, the limited number of studies, the small number of patients and the limited studies in adults cause low to very low overall quality of evidence. All studies experienced side effects such as short-term gastrointestinal upset, high cholesterol, and other [6,7]. The study times were short, so the long-term risk associated with these adverse effects is unknown. Only few studies used the ketogenic diet in adults with drug-resistant epilepsy; therefore, further studies could be useful. For people with difficult-to-treat epilepsy or people who are unsuitable for surgical intervention, a ketogenic diet remains an important option; however, further research is needed regarding this issue. Future randomized clinical trials are needed to confirm the efficacy of the ketogenic diet in various types of epilepsy and to provide further information on some unresolved practical problems, i.e., how long the ketogenic diet should be continued.

It was noted that the susceptibility and frequency of seizures increased in animals on a ketogenic diet after administration of high doses of antibiotics, which led to exhaustion of the microbiota [40]. The negative effect of antibiotic therapy was reversed when the intestine was re-colonized with bacteria. The diversity of the intestinal microbiota has decreased, while the relative numbers of *Akkermansia muciniphila* and *Parabacteroides merdae* have increased during the ketogenic diet, so these specific changes may play a role in anti-seizure activities. This review deepens our knowledge about the effects of intestinal microflora on brain function and behavior. The work reveals the causal role of microflora in mediating the anticonvulsant action of the ketogenic diet in mice and shows the molecular and cellular pathways that may be involved, as a result of which interactions between selected bacteria regulate peripheral metabolites that affect the levels of neurotransmitters in the hippocampus [40]. It should be noted that additional research is needed to determine if similar effects on amino acids and brain metabolites occur in humans following a ketogenic diet. In addition, although the current study has included the hippocampus in these processes, there is little evidence from previous studies suggesting that it plays an important role in childhood epilepsy. The effect of ketogenic diet on health and disease is promising, but much more research needs to be done. The positive effect of ketogenic diet is achieved through a number of mechanisms that among others lead to reduced excitability of neurons, as well as through changes in the intestinal microflora. All the papers selected to illustrate the crossing mechanisms revealed the alleged links between intestinal microbiome, ketogenic diet, and systemic effects. Some findings are demonstrated by amino acids analysis, some are only a conjecture. As can be seen, there are many controversial findings suggesting the need for a deeper understanding of the problem. Observations that the ketogenic diet can modulate and transform the intestinal microflora create a potential and promising basis for future diet therapy. The ketogenic diet has been shown to be a powerful tool and requires further refinement and good formulation, given its effect on intestinal health. In conclusion, further studies of long-term clinical trials should be carried out to establish safer and healthier dietary interventions for patients.

Further experimental study has documented unexpectedly that mice kept on the ketogenic diet exhibited proconvulsant effects in electroshock-induced seizures. This result was probably due to the fact that pronounced metabolic reserves (actually produced by the diet) were necessary for the spread of electroconvulsions throughout the central nervous system [47]. However, the main ketone body, acetone, produced in the course of the ketogenic diet, elevated significantly the threshold for electroconvulsions in mice [48]. In addition, acetone very significantly enhanced the anticonvulsant activity of valproate against maximal electroshock-induced convulsions in mice, other antiepileptic drugs (carbamazepine, lamotrigine, phenobarbital) being also, but less significantly, potentiated. As regards oxcarbazepine, phenytoin, and topiramate—they were not affected by acetone at all. Importantly, the adverse effects of antiepileptic drugs were not increased by acetone [48]. Clinical data also point to valproate as an antiepileptic drug very positively interacting with the ketogenic diet [49]. 

The use of a ketogenic diet gives the possibility of treating drug-resistant epilepsy with proven effectiveness. Over the past few years, variants of the ketogenic diet have been developed to make therapy easier and tastier, while reducing side effects and making it available to a larger group of patients with refractory epilepsy. Clinical evidence suggests that a rigid and strict initiation protocol with carbohydrate restriction and increased fat intake is the key to achieving high efficacy. The choice of the diet must be made on an individual basis considering the patient’s age, family circumstances, and severity and type of epilepsy. Pediatric and adult neurologists must be able to identify and refer appropriate patients to the ketogenic diet as soon as necessary in the course of epilepsy instead of using it as a last option only.

## 8. Outlook

Recent studies have shown a close relationship between epilepsy and the intestinal microbiome. Furthermore, the intestinal microbiome may be a mechanism to prevent seizures treated with a ketogenic diet. There is therefore great potential in optimizing the ketogenic diet to promote certain microorganisms as neuroprotective treatment of drug-resistant epilepsy. Nevertheless, many questions still need to be fully answered, including the particular mechanism by which the intestinal microflora affects the onset of epilepsy and the possible clinical applications of discoveries related to the function of the intestinal microflora in the treatment by the ketogenic diet epilepsy. Moreover, these questions can only be answered by undertaking large, multi-center research efforts.

To date, the results of using the ketogenic diet as a drug obtained in the treatment of drug-resistant epilepsy seem to be particularly interesting in connection with the reduction of seizure frequency and cognitive function improvement [50], despite the limited number of studies. The few human studies available to date are based on a pre/post design, but without an authentic reference control group and randomization. The results showed causal evidence and underline the need to increase the number of studies to demonstrate that the ketogenic diet reduced seizure activity and produced cognitive improvement [50]. In recent years, the reputation of the ketogenic diet in terms of its therapeutic effects is clearly growing. In molecular studies and partly polemic publications, the ketogenic diet gives misleading results because this diet does not have single target point [6,7,8]. In addition, the fact that the ketogenic diet, like many other natural substances, has more than one drug target, indicates its versatile use and low risk of inducing resistance to treatment. Although there are reasonable doubts, essential to the reliability of therapeutic results, it makes no sense to disparage everything that has been published so far about the therapeutic effects of the ketogenic diet in the treatment of neurodegenerative diseases, malignant gliomas, or drug-resistant epilepsy [8]. Instead, we should take the challenge of distinguishing between scientifically substantiated and false treatment data; otherwise, we will lose the promising diet necessary for complementary and alternative therapy strategies in the case of drug-resistant epilepsy. Rejecting some of the effects of ketogenic diet therapy would simply be ignorance. Opponents of the ketogenic diet criticize the fact that it has never been demonstrated to be definitely effective in a randomized, double-blind, placebo-controlled clinical trial [8]. Critics should consider the fact that it is almost impossible to get financial support for conducting a clinical trial with a substance/diet that cannot be patented and that will not bring economic benefits in the future. Another issue to consider is the design of the study, the ketogenic diet cannot be tested in randomized placebo-controlled studies, since clinical trials are currently required to compare the test substance with standard therapy, otherwise the study will not get approval from the ethics committee. Therefore, the question is against which drug now used to treat epilepsy should we test the ketogenic diet? There is no doubt that due to comprehensive data from preclinical studies, as well as the first results of individual patients or small cohorts, the next task must be to test the ketogenic diet in well-designed and control clinical trials. However, the biggest challenge will be finding sponsors for clinical research on the ketogenic diet, because this promising diet will not bring economic benefits to potential sponsors. Despite this, further double-blind studies are needed to clarify the effectiveness of the ketogenic diet. In conclusion, future research should focus on the correct identification of patients. Further assessment of the specifics of the mechanisms may contribute to the replacement of strict treatment by ketogenic diet with dietary supplements such as probiotics and/or prebiotics [51]. Moreover, the exact and definitive explanation of ketogenic diet therapy can give hope for a long-term therapeutic effect that can be continued even after the diet has been ended through dietary supplements.
